# Chemical Affinity of Ag-Exchanged Zeolites for Efficient Hydrogen Isotope Separation

**DOI:** 10.1021/acs.inorgchem.2c00028

**Published:** 2022-06-14

**Authors:** Linda Zhang, Toshiki Wulf, Florian Baum, Wolfgang Schmidt, Thomas Heine, Michael Hirscher

**Affiliations:** † Max Planck Institute for Intelligent Systems, Heisenbergstr. 3, 70569 Stuttgart, Germany; ‡ Wilhelm-Ostwald-Institut für Physikalische und Theoretische Chemie, Linnéstraße 2, 04103 Leipzig, Germany; § Helmholtz-Zentrum Dresden-Rossendorf, Forschungsstelle Leipzig, Permoserstraße 15, 04318 Leipzig, Germany; ∥ Department of Heterogeneous Catalysis, Max-Planck-Institut für Kohlenforschung, Kaiser-Wilhelm-Platz 1, 45470 Mülheim an der Ruhr, Germany; ⊥ Fakultät für Chemie und Lebensmittelchemie, TU Dresden, Bergstraße 66c, 01062 Dresden, Germany

## Abstract

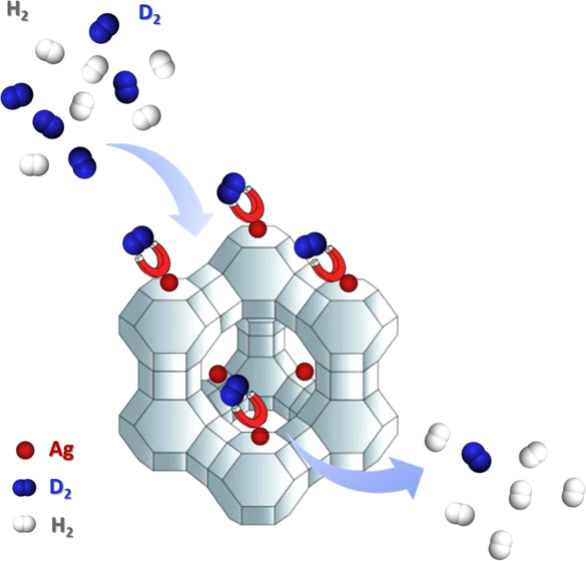

We report an ion-exchanged zeolite as an excellent candidate for large-scale application in hydrogen isotope separation. Ag(I)-exchanged zeolite Y has been synthesized through a standard ion-exchange procedure. The D_2_/H_2_ separation performance has been systematically investigated via thermal desorption spectroscopy (TDS). Undercoordinated Ag^+^ in zeolite AgY acts as a strong adsorption site and adorbs preferentially the heavier isotopologue even above liquid nitrogen temperature. The highest D_2_/H_2_ selectivity of 10 is found at an exposure temperature of 90 K. Furthermore, the high Al content of the zeolite structure leads to a high density of Ag sites, resulting in a high gas uptake. In the framework, approximately one-third of the total physisorbed hydrogen isotopes are adsorbed on the Ag sites, corresponding to 3 mmol/g. A density functional theory (DFT) calculation reveals that the isotopologue-selective adsorption of hydrogen at Ag sites contributes to the outstanding hydrogen isotope separation, which has been directly observed through cryogenic thermal desorption spectroscopy. The overall performance of zeolite AgY, showing good selectivity combined with high gas uptake, is very promising for future technical applications.

## Abstract 2

Silver-exchanged zeolite Y was synthesized for efficient and effective separation of hydrogen isotopes above liquid nitrogen temperature via chemical affinity sieving. The highest D_2_/H_2_ selectivity of 10 is achieved at 90 K combined with a high gas uptake, making zeolite AgY a promising candidate for large-scale deuterium enrichment.

## 1 Introduction

As a stable isotope of hydrogen, deuterium plays an irreplaceable role in numerous industrial and scientific applications, such as nonradioactive tracing, neutron scattering, neutron moderation, and pharmaceutics.^1−6^ In addition, deuterium is recognized as a fuel component for nuclear fusion reactors, one of the most promising alternatives for future clean energy supply.^7,8^ Although hydrogen is the simplest and most abundant element in the universe, deuterium only represents 0.0156 mol % of all hydrogen on earth. Therefore, large-scale separation of light isotopes is required to ensure the reliable supply of this key material. In industry, cryogenic distillation, based on the difference in the boiling temperatures of hydrogen isotopes (20.37 K for H_2_, 23.71 K for D_2_), is still one of the most common separation methods for hydrogen isotopes. However, this approach is energy-intensive and shows a low separation factor (∼1.5).^9,10^ Because of the significantly high cost of this technology, other processes that can efficiently separate hydrogen isotopes at higher temperatures are highly demanded.

One of the most promising alternatives for hydrogen isotope separation is based on physisorption on nanoporous materials. Molecules with a strong chemical affinity toward the surface in the pores are generally adsorbed, while those exhibiting weaker interaction are not. Deuterium can be preferentially adsorbed via chemical affinity sieving (CAS)^11^ on strongly binding sites of the host materials, mainly unsaturated metal centers. Owing to the lower zero-point energy (ZPE), the heavier isotope interacts much stronger with the adsorbent, leading to a higher adsorption enthalpy (Δ*H*). FitzGerald et al.^12^ first explored a CAS effect in MOF-74 with various open metal sites (Fe, Co, and Ni). A D_2_/H_2_ selectivity of 5 was observed at 77 K, demonstrating that efficient separation can be achieved above liquid nitrogen temperature. Similarly, Oh et al.^13^ reported an outstanding experimental value of 11.8 for D_2_/H_2_ selectivity at 60 K in CPO-27-Co. Even at a temperature of 80 K, i.e., above liquid nitrogen, CPO-27-Co still exhibited an impressive selectivity of 6.3. When undercoordinated Cu(I) sites were introduced into MFU-4l, a large difference in the adsorption enthalpy of 2.5 kJ/mol between D_2_ and H_2_ was observed, resulting in a selectivity of 11 at 100 K.^14^ Based on the previous studies, strong adsorption sites attract the heavier isotopes at relatively high temperatures, resulting in an energy-efficient separation approach with high selectivity. However, the low density of strong adsorption sites on metal clusters, as well as the costly synthesis process, prevents MOFs to be used in large-scale applications. On the other hand, zeolites are widely used porous materials in many large-scale applications because of their merits including low cost, excellent thermal stability, radiation resistance, and well-defined pore structure.^15−17^ Therefore, ion-exchanged zeolites are considered potent candidates for hydrogen isotope separation. Until now, many ion-exchanged zeolites have been experimentally studied for hydrogen isotope separation.^18−23^ Bezverkhyy et al.^24^ studied the D_2_/H_2_ adsorption selectivity on K–Na-exchanged zeolite A, and a selectivity of 23 has been obtained at a temperature of 48 K due to the accessibility of D_2_ only to the α cage of zeolite A. Xiong et al.^25^ have reported that unsaturated Cu(I) sites in ZSM-5 exhibited a selectivity of 26 at 100 K for D_2_ over H_2_, which is one of the highest values ever reported. Unsaturated Ag has also been studied in ZSM-5 by Xiong et al.,^26^ with a D_2_ over H_2_ selectivity of 8.7 at 77 K. However, the low density of exchanged ions in ZSM-5 limits the adsorption capacity and therefore makes these reported zeolites not an ideal solution for practical applications.

Based on previous studies, an ideal zeolite for hydrogen isotope separation should process a substantial density of strong adsorption sites, which can preferentially attract deuterium even at high temperatures. Herein, we report highly efficient and effective hydrogen isotope separation using an ion-exchanged zeolite Y with high uptake and a D_2_ over H_2_ selectivity of 10 at 90 K through Ag(I) incorporation. Thermal desorption spectroscopy (TDS) is employed to determine the hydrogen isotope separation performance. The isotope effect has been confirmed further based on theoretical calculations.

## 2 Experimental Section

### 2.1 Syntheses of Ag-Exchanged Zeolites

Zeolite NaY (AlSiPenta GmbH, now Clariant) was used as the starting material. The AgY zeolite was obtained by ion-exchanging 1 g of NaY in 125 mL of 0.1 M AgNO_3_ solution (2.12 g of AgNO_3_ dissolved in 125 mL of deionized argon-saturated water) for 2 h under an argon atmosphere in a vessel covered with aluminum foil to shield the solution from exposure to light. After 2 h, the sample was separated by vacuum filtration and washed with argon-saturated deionized water. Then, the whole procedure was repeated another two times to achieve a quantitative exchange of Na by Ag. The final AgY was then dried at 80 °C for 12 h and stored in the absence of light.

### 2.2 Characterizations

The XRD pattern of NaY was measured in sealed 0.5 mm borosilicate glass capillaries with a Stoe STADI P transmission diffractometer equipped with a bent Ge monochromator and a position-sensitive detector (Stoe). The XRD pattern of AgY was measured in sealed 0.5 mm borosilicate glass capillaries with a Rigaku SmartLab diffractometer equipped with a 9 kW rotating anode, a Johannson monochromator, and a HyPix3000 2D detector. The capillary with AgY was kept in the absence of light prior to the measurement. Chemical analysis of a dissolved portion of the sample was performed with a Spectrogreen ICP OES spectrometer (Spectro). TG/DSC data were measured with an STA 449 F3 thermobalance (Netzsch) at a heating rate of 10 K/min by applying a constant argon flow. The instrument was coupled to an Aeolos QMS mass spectrometer (Netzsch).

### 2.3 TDS Studies of Hydrogen Isotope Separation

The D_2_/H_2_ isotope separation measurements were performed using an in-house designed setup for thermal desorption spectroscopy (TDS).^27^ Before the measurements, the sample was activated inside the TDS device under high vacuum (10^–5^ mbar) at 500 K for 2 h. Then, the sample was exposed to the atmosphere of D_2_ and/or H_2_ at a fixed temperature (*T*_exp_) for a chosen exposure time. The nonadsorbed gas was evacuated by a turbomolecular pump, and then, the sample was cooled down to 20 K to retain this adsorbed state. Finally, the desorbed D_2_/H_2_ molecules were measured using a quadrupole mass spectrometer (MKS, Microvision plus) while heating at a rate of 0.1 K/s up to room temperature.

### 2.4 Density Functional Theory (DFT) Calculations of D_2_/H_2_ Separation

The framework structure of FAU has been obtained from the Database of Zeolite Structures from the International Zeolite Association.^28^ A model cluster has been cut out of the structure with one T site as Al, all remaining T sites as Si, dangling O–Si groups replaced with O–H groups, the direction of the bonds from the original structure preserved, and the O–H bond length set to 95 pm. Geometry optimizations with Cartesian constraints on the terminal O–H groups and ten different positions for the Ag atom (corresponding to the center of the faces and edges of the AlO_4_ tetrahedron) have been carried out, yielding six unique positions. For each of the five positions with undercoordinated Ag^+^, the model cluster has been cut down further to enhance the computational efficiency of the ensuing steps.

Where water molecules have been placed into the cluster, this has been done incrementally: For each additional step, we have targeted a Ag–O distance of approximately 230 pm, that the H atoms face framework O atoms (or O atoms of already adsorbed water molecules) at a distance of at least 280 pm and at most 600 pm and no starting structure with an interatomic distance of less than 150 pm between any of the atoms of the water molecule and any of the framework atoms has been allowed. Clusters with water have been optimized with Cartesian constraints on the terminal O–H groups of the framework only. The hydrogen molecule has been placed at a distance of 182 pm to the Ag atom at the position with the least steric hindrance, and constrained geometry optimization has been carried out with the same constraints as in the previous steps.

All geometry optimizations have been carried out at the hybrid density functional level of theory employing the PBE0^29^ functional with Grimme’s D3 empirical dispersion correction and Becke–Johnson damping, commonly referred to as D3(BJ).^30^ Ahlrich’s def2-TZVP basis set^31^ has been employed in conjunction with the def2/J auxiliary basis set^32^ for the resolution-of-identity and chain-of-spheres approximation (RIJCOSX).^33^ Vibrational and thermodynamic properties of adsorption have been determined using the harmonic approximation of the vibrational modes of the H_2_ molecule in the field of the constrained framework atoms. For a generic zeolite model,^34^ this approach has been validated against vibrational analysis constraining only the terminal O–H groups (see the Supporting Information).

Correlated single-point energy calculations have been carried out using the DLPNO-CCSD(T) method^33^ as implemented in the ORCA program system^35,36^ employing NormalPNO cutoff thresholds^37^ and the def2-TZVPP basis.^38^ For a subset, calculations with the larger def2-QZVPP basis set have been carried out and found to yield nearly identical results. Thermodynamic properties have been calculated based on correlated single-point energies and density functional level vibrations.

## 3 Results and Discussion

### 3.1 Characterization of Zeolite AgY

**Figure 1:**
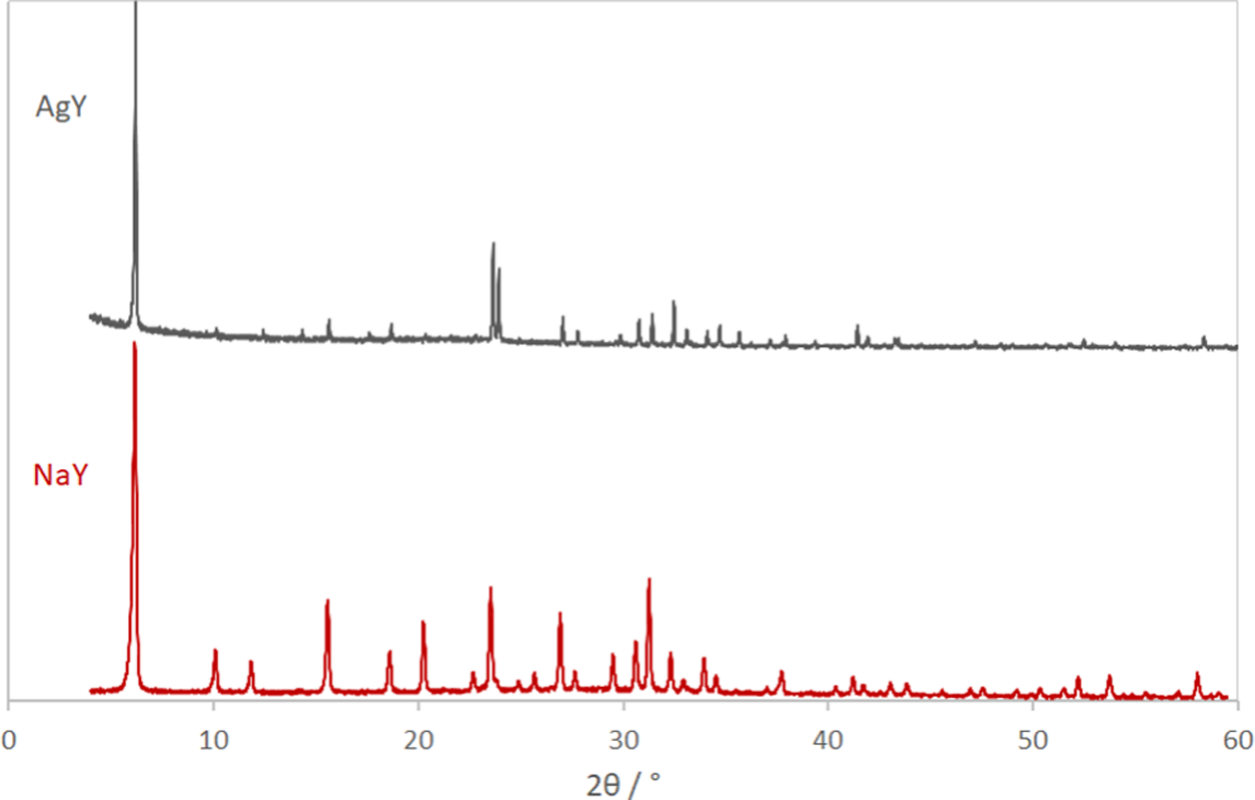
XRD patterns of NaY (Stoe STADI P) and AgY (Rigaku SmartLab).

The XRD pattern of AgY in Figure 1 shows that the zeolite Y structure is well preserved after ion exchange. In comparison to the XRD pattern of parent NaY, relative reflection intensities changed as expected due to the replacement of Na^+^ by Ag^+^ with a much higher electron density. Both samples were measured in capillaries in the transmission mode. However, due to the strong absorption of X-rays by the silver in AgY, the sample needed to be measured on an XRD instrument (Rigaku SmartLab) equipped with a rotating anode, which provides a much higher intensity of the X-ray beam than the conventional transmission diffractometer (Stoe STADI P). The ion-exchanged zeolite had a Si/Al ratio of 2.57 and a Ag/Al ratio of 1.04 as determined by ICP OES. The residual sodium content of the sample was below the detection limit of 2.6 ng/g. The water content of the ion-exchanged zeolite was 15.14 wt % as determined by TG/DSC measurements. This indicated that a small amount of silver is deposited also on the external surface of the zeolite, likely as amorphous silver oxide, since no reflections of a crystalline by-phase are visible in the XRD pattern. We were aware of the fact that autoreduction of Ag^+^ upon thermal dehydration under vacuum will result in a certain amount of Ag(0) and may be even smaller clusters within the zeolite. However, in that process, water will be oxidized to O_2_, and QMS data measured during heating in argon in the TG/DSC instrument indeed show that O_2_ is released from the sample. As the signal (*m*/*z* = 32) observable is just about the detection limit of the QMS (see Figure S1), we assumed that only a fraction of Ag^+^ is reduced to Ag(0).

### 3.2 Cryogenic TDS Experiments on Single Gases

**Figure 2:**
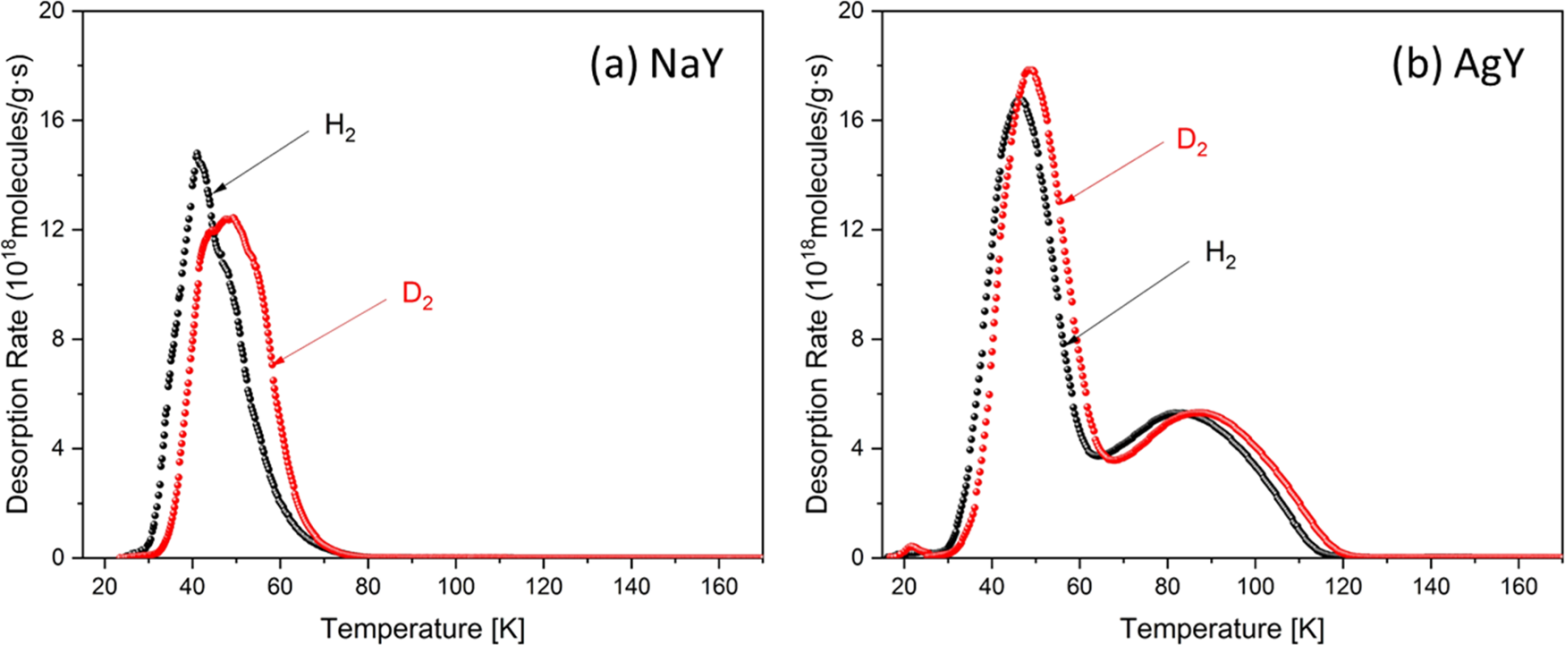
Thermal desorption spectroscopy (TDS) curves for (a) NaY zeolite and (b) AgY zeolite after exposure to 10 mbar of pure H_2_ and D_2_ gas at room temperature followed by cooling to 20 K.

Pure H_2_ and D_2_ cryogenic TDS measurements were performed to identify the preferential adsorption sites on Na- and Ag-exchanged zeolite. At room temperature, the samples were exposed to 10 mbar of H_2_ or D_2_ under identical experimental conditions, and then, the samples were cooled to 20 K. Figure 2 shows the H_2_ and D_2_ desorption curves in the temperature range of 15–165 K, at a heating rate of 0.1 K/s. The desorption curves for NaY and AgY exhibit in the low-temperature regime a desorption peak centered at 45 and 50 K for hydrogen and deuterium, respectively. By contrast, only zeolite AgY shows an additional distinct desorption peak at higher temperatures, with maxima at 82 and 87 K for H_2_ and D_2_, respectively. With increasing temperature, TDS measurements represent typically sequential desorption from weak to strong adsorption sites. The two desorption maxima can be assigned to two different adsorption sites in the framework of zeolite AgY possessing different adsorption energies. The first desorption maximum below 60 K corresponds to pore filling because the adsorption enthalpies of H_2_ and D_2_ are comparatively weak. The second maximum above 60 K is attributed to strong adsorption sites with a higher enthalpy, *i.e.*, undercoordinated Ag(I). Here, the desorption maximum of D_2_ is slightly shifted to a higher temperature in comparison to that of H_2_, as evident by a slightly higher adsorption enthalpy for D_2_ than for H_2_. The temperature shift for D_2_ interacting with the Ag^+^ cation is more pronounced than that for H_2_, revealing stronger interaction of D_2_. The integrated area under the desorption curve is directly proportional to the number of adsorbed molecules and can be quantified after calibration with Pd_95_Ce_5_ alloy (Supporting Information). Zeolite NaY exhibits an isotopologue-independent uptake for H_2_ and D_2_ with 6.3 mmol/g, whereas for zeolite AgY, the total amounts of H_2_ and D_2_ are 8.8 and 9.3 mmol/g, respectively. The exceeding uptake of about 3 mmol/g is related to the high-temperature desorption peak.

### 3.3 Experimental D_2_/H_2_ Separation

**Figure 3:**
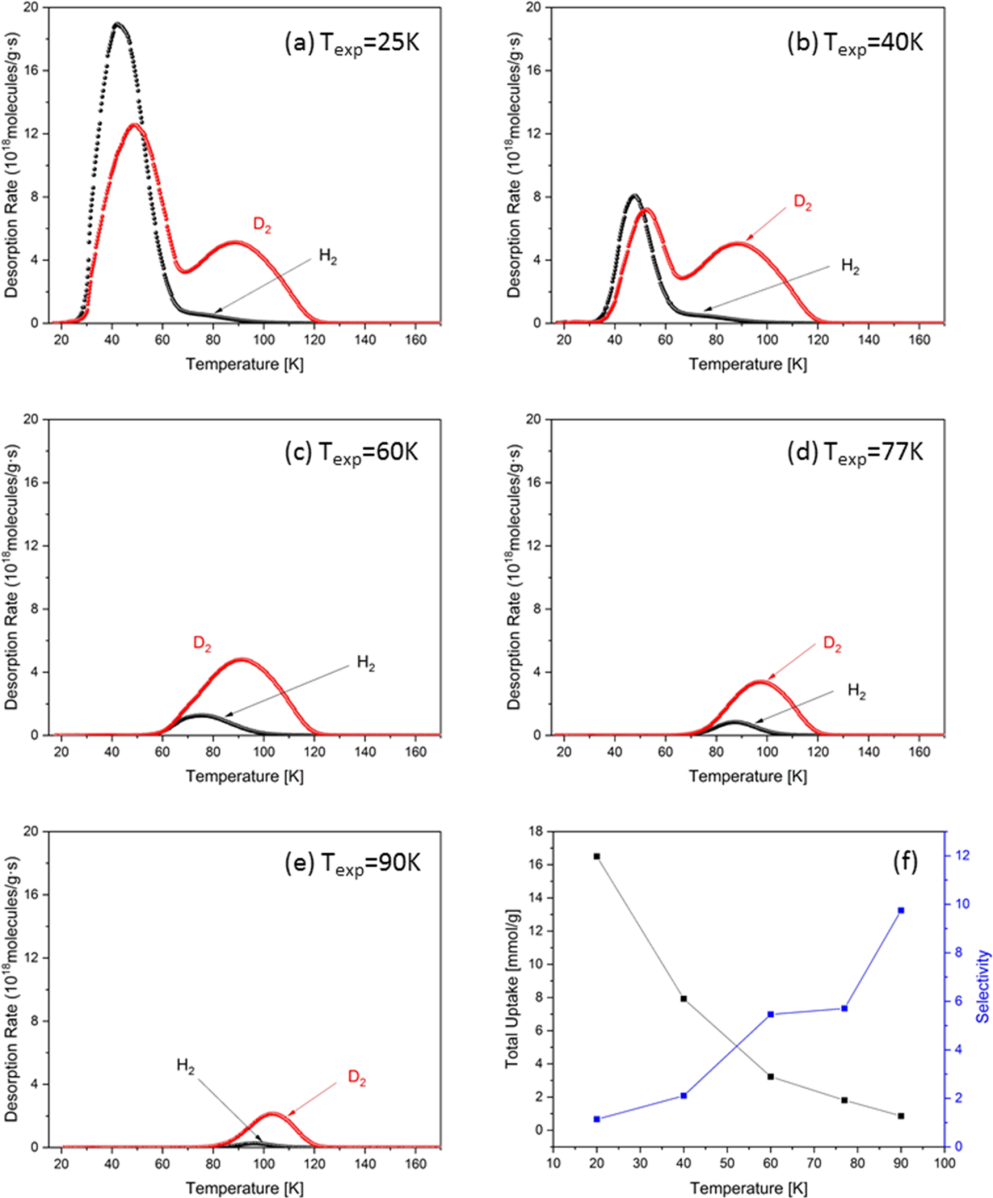
H_2_ (black) and D_2_ (red) desorption curves after adsorption of a 1:1 D_2_/H_2_ mixture exposed at 10 mbar to zeolite AgY at temperatures of (a) 25 K, (b) 40 K, (c) 60 K, (d) 77 K, and (e) 90 K. (f) Selectivity and the corresponding amount of adsorbed D_2_ as a function of adsorption temperature.

The desorption curves of H_2_ and D_2_ in zeolite AgY, measured after adsorbing a 1:1 D_2_/H_2_ isotope mixture (10 mbar) at different *T*_exp_’s of 25, 40, 60, 77, and 90 K, are presented in Figure 3a–e. All desorption curves show nearly no gas release below the exposure temperature since during the evacuation process carried out at the same temperature all more weakly bound molecules have been already removed. When the exposure temperature is lower than 40 K, two desorption maxima can be detected, pointing to at least two adsorption sites in the framework possessing different binding enthalpies. The desorption peaks for H_2_ and D_2_ primarily occurring below 60 K are ascribed to weak adsorption sites in the zeolite supercages; in contrast, the ones above 60 K are attributed to molecules released from undercoordinated Ag sites. As expected, only one desorption maximum can be observed with increasing exposure temperatures. After exposure to an equimolar mixture, these strong sites are predominately occupied by D_2_, as shown by the remarkably higher maximum rate in the D_2_ desorption curve compared with that of H_2_, implying high D_2_/H_2_ selectivity at Ag sites. The variation of D_2_/H_2_ selectivity and the corresponding amount of D_2_ adsorbed in the framework as a function of exposure temperature are shown in Figure 3f. At *T*_exp_’s lower than 40 K, *S*_D_2_/H_2__ is below 2 because of the weak adsorbent–adsorbate interaction; on the contrary, when the *T*_exp_ increases above 60 K, due to the energetically favored binding sites, *S*_D2/H2_ considerably increases and reaches a maximum of 10 at an exposure temperature of 90 K. Even the total gas uptake is reduced at increasing exposure temperature, the adsorbed amount of D_2_ remains still around 1 mmol/g. Herewith, AgY exhibits one of the best combinations between selectivity and adsorbed amount at an operating temperature above the boiling point of liquid nitrogen, therefore having high potential for practical isotope separation application.

Another important observation reveals the isotope exchange on Ag sites. Figure S3 presents the TDS curves of zeolite AgY collected after exposure to an equimolar D_2_/H_2_ mixture at various pressures of 1–100 mbar for 10 min at 60 K. At a p_exp_ of 1 mbar, the D_2_ over H_2_ selectivity is close to 1 (1.24 mmol/g for D_2_ and 1.05 mmol/g for H_2_). On the Ag adsorption sites, D_2_ and H_2_ show nearly equal occupancy. The number of metal sites is large enough to adsorb all D_2_ and H_2_, allowing simultaneous occupation without competition. At higher exposure pressures (*p*_exp_ ≥ 5 mbar), the area under the D_2_ desorption curve is remarkably surpassing that of H_2_, indicating that D_2_ preferentially occupies the open metal sites, resulting in high selectivity. Moreover, the amount of the adsorbed gas increases with higher exposure pressure (Figure S4). As *p*_exp_ increases from 1 to 10 mbar, the total amount of the adsorbed gas (H_2_ plus D_2_) increases from 2.2 to 3.4 mmol/g. The total amount remains constant upon further increase of the exposure pressure; however, the selectivity keeps increasing with increasing p_exp_ above 10 mbar. Similar phenomena can be observed at different exposure times. TDS curves collected after exposure to a 1:1 isotope mixture for 1–120 min are shown in Figure S5. The uptake of the isotopes and the D_2_/H_2_ selectivity as a function of exposure time are presented in Figure S6. For longer exposure times, the adsorbed amount of H_2_ decreases, whereas that of D_2_ increases. Since the amount of total gas adsorbed remains nearly constant at 3.2 mmol/g, consequently, the selectivity will increase from 3.9 to 6.3. Furthermore, this increase in selectivity can only be explained by a replacement of H_2_ with D_2_ at Ag sites with increasing exposure time. A similar effect was previously identified for hydrogen isotopes in Cu(I)-MFU-4l, a MOF containing strong open metal sites.^14^ The highest amount of adsorbed gas is ∼3.25 mmol/g in terms of measurements either at higher pressures or longer exposure times, suggesting that at 60 K all accessible adsorption sites are fully occupied and, under the assumption of one gas molecule per Ag site, these numbers are equal. This is in accordance with a value of 3.17 mmol/g Ag in dry AgY (corresponding to 34.2 wt % Ag and Si/Al of 2.62).

Based on the thermal desorption curves, desorption energies were determined by employing the Kissinger method.^39^ Under the assumption of a fully reversible adsorption and desorption process and desorption energy independent of coverage and temperature, Kissinger plots provide the Arrhenius energy of activation for the desorption process. Since no transition state is expected to exist, this energy is expected to be similar to the energy of desorption. Desorption curves for H_2_ and D_2_ from zeolite AgY were measured after loading the two isotopes at *T*_exp_ = 60 K. For the measurements, various heating rates (0.2, 0.1, 0.05, and 0.01 K/s) were applied and the subsequent desorption curves are displayed in Figure S5. The desorption maximum shifts to higher temperatures for higher heating rates, indicating that the isotope desorption process is thermally activated. For these silver sites, the desorption energies for H_2_ and D_2_ as calculated from the Kissinger plots (Figure S8) are 9.1 and 11.7 kJ/mol, respectively. The heavier D_2_ has higher desorption energy than H_2_ by 2.6 kJ/mol, which explains the enhanced selectivity for the two isotopes.

### 3.4 DFT Calculations of D_2_/H_2_ Separation on Silver

The TDS measurements and their analyses indicate that the hydrogen isotopes are preferentially adsorbed on silver cations in the framework, which are expected to exhibit the strongest chemical affinity quantum sieving due to the highest ZPE difference between adsorbed H_2_ and D_2_ molecules. For a deeper understanding of the phenomenon of isotopologue-selective adsorption of hydrogen at silver sites, a finite structure model has been applied to simulating the molecule–framework interaction. The construction of the finite molecular model is described in the Experimental Section. All calculations have been carried out utilizing London dispersion-corrected density functional theory (DFT) as described there.

#### 3.4.1. Position of Silver Cations

DFT calculations show six distinct equilibrium positions for the Ag^+^ cation within the zeolite. The most stable ones—well-known cation sites in faujasitic zeolites^40^—are shown in Figure S8. In one of these, the Ag^+^ cation is coordinatively saturated inside the hexagonal prism (site I). Since this position does not allow for H_2_ adsorption, it has not been investigated further. Of the structures with undercoordinated Ag^+^, the most stable structure has the cation located at the open six-ring of the sodalite cage (site II) in agreement with Ag^+^ sitting in FAU-type zeolites.^41^ The position at the interface between a hexagonal prism and a sodalite cage (site I′) is predicted to be approximately 30 kJ·mol^–1^ less stable and according to the literature may or may not be occupied depending on the activation conditions. The three remaining sites are predicted to be less stable by another 20–30 kJ·mol^–1^ and have not been observed experimentally in zeolite AgY.^42^ We therefore exclude them from further analysis as well.

#### 3.4.2. H_2_ Adsorption Enthalpy and Gibbs Free Energy Assuming Water-Free Zeolite

Table 1 shows the adsorption enthalpies and Gibbs free energies of adsorption of H_2_ and D_2_ calculated using atomistic models of Ag^+^ located at sites II and I′. Unsurprisingly, less stable site I′ is also more active and adsorbs both isotopologues more strongly. At 90 K, both sites are predicted to have a D_2_ adsorption enthalpy that is more attractive by 2.3 kJ·mol^–1^—a difference that is dominated by the zero-point energy; thermal contributions are negligible. Due to entropy contributions, the difference between the Gibbs free energies of adsorption is notably smaller with 1.6 kJ·mol^–1^ in favor of D_2_. This fight against entropy is a general observation for chemical affinity quantum sieving and the reason why it is so difficult to scale to higher temperatures.

**Table 1:** Ag^+^ Attachment Energy, Difference in the Zero-Point Energy of Adsorption between H_2_ and D_2_, H_2_ and D_2_ Adsorption Enthalpies (*T* = 90 K, *p* = 10 mbar), and Gibbs Energies (kJ·mol^–1^) at Sites II and I′ Calculated Using DFT and Nonperiodic Models with a Single Ag^+^ Ion

site	Δ_ad_*E*(Ag)	ΔΔ_ad_*E*_zp_	Δ_ad_*H*(H_2_)	Δ_ad_*H*(D_2_)	Δ_ad_*G*(H_2_)	Δ_ad_*G*(D_2_)	*S*(D_2_/H_2_)
II	–660.2	–2.4	–17.7	–20.1	–6.9	–8.5	8.0
I’	–628.0	–2.3	–23.4	–25.7	–12.7	–14.2	7.5

#### H_2_ Adsorption Enthalpy and Gibbs Free Energy Assuming the Presence of Water

**Figure 4:**
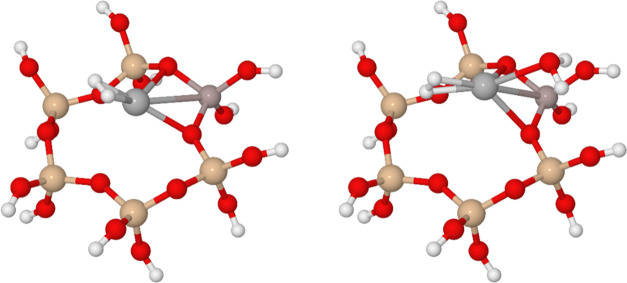
H_2_ adsorbed on Ag^+^ at site II; without (left) and with one molecule of water (right). Unlike H_2_, which seeks to minimize interaction with peripheral framework atoms, H_2_O seeks this interaction; due to these different preferences, H_2_O adsorption does not necessarily hinder subsequent adsorption of H_2_. Beige, Si/Al; red, oxygen; gray, silver; white, hydrogen.

Table 2 shows the thermodynamic properties of Ag^+^ sites that coordinate a single water molecule. As expected, the presence of water significantly diminishes enthalpies and especially the Gibbs free energies of adsorption. However, our calculations still predict a significant attraction and a D_2_/H_2_ selectivity that is almost unchanged for more stable site II. The former can be understood when taking into consideration the different adsorption properties of water and hydrogen, as shown in Figure 4. While hydrogen interacts strongly only with the Ag^+^ site and seeks to minimize interaction with atoms in the periphery, water not only binds to the Ag^+^ site via its O atom but also can significantly stabilize itself by forming hydrogen bonds with peripheral framework atoms. This behavior can leave enough room for subsequent adsorption of hydrogen. However, apart from the positions elaborated in Table 2, other positions for the water molecule are possible and they result in lower adsorption enthalpies. Because entropy contributions are significant, none of these positions are predicted to adsorb the hydrogen isotopologues at 90 K. After adsorption of a second water molecule, hydrogen will not adsorb at Ag^+^ at site II or I′ anymore.

**Table 2:** H_2_O Adsorption Energies, Subsequent H_2_ and D_2_ Adsorption Enthalpies (*T* = 90 K, *p* = 10 mbar), and Gibbs Energies (kJ·mol^–1^) at Sites II and I′ Calculated Using DFT and Nonperiodic Models with a Single Ag^+^ Ion

site	Δ_ad_*E*(Ag) + Δ_ad_*E*(H_2_O)	ΔΔ_ad_*E*_zp_	Δ_ad_*H*(H_2_)	Δ_ad_*H*(D_2_)	Δ_ad_*G*(H_2_)	Δ_ad_*G*(D_2_)	*S*(D_2_/H_2_)
II	–732.5	–2.5	–13.5	–16.0	–2.6	–4.2	8.9
II	–728.8	–2.6	–18.4	–21.0	–7.4	–9.1	9.3
I′	–713.8	–1.9	–10.4	–12.3	+0.4	–0.6	4.1

We use a finite (cluster) model system with a single Ag^+^ atom to model the adsorption of H_2_, thereby restricting ourselves to the Langmuir regime. Therefore, pressures have to be reasonably low and temperatures should be significantly above the boiling temperature of hydrogen. The cluster model in conjunction with the static harmonic vibrational calculations that we use cannot account for the anharmonicity of the vibrational modes and the dynamic behavior of the zeolitic systems. We expect the harmonic approximation to slightly overestimate the vibrational enthalpy contributions by approximately 5–10% of the zero-point energy, resulting in a difference of less than 0.3 kJ·mol^–1^ for the isotopologue selectivity. Given these fundamental challenges, we make further approximations in the treatment of vibrational modes, as outlined in the Supporting Information. While they strongly reduce the complexity of the calculations, we conclude that they do not lead to significant additional errors in the results given that their expected effect on the difference between the desorption energies of D_2_ and H_2_ is calculated to be on the order of 0.1 kJ·mol^–1^.

## 4 Conclusions

In conclusion, we experimentally investigated the D_2_/H_2_ separation on silver-exchanged zeolite Y. Efficient hydrogen isotope separation can be achieved due to the stronger interaction of D_2_ with Ag^+^ cations within the zeolite micropores. Direct D_2_-over-H_2_ separation experiments were carried out by thermal desorption spectroscopy using a 1:1 isotope mixture. The highest selectivity of 10 at a *T*_exp_ of 90 K can be observed due to the chemical affinity sieving at strong adsorption Ag^+^ sites. Based on the results obtained from theoretical analysis, the large isotope effect is attributed to the large difference in the zero-point energy, leading to the large difference in the adsorption enthalpy of 2.6 kJ/mol between H_2_ and D_2_, which is in accordance with the experimental result. Moreover, in contrast to previously studied MOFs, such as CPO-27-Co and Cu(I)-MFU-4*l*, this material shows a higher mass density of strong adsorption sites, offering a higher adsorption capacity for D_2_. Thus, with a high selectivity above liquid nitrogen temperature, AgY presents itself as a promising candidate for large-scale deuterium enrichment.

## Supporting Information Available

Additional characterization of the sample, details of experimental TDS measurements, detailed evaluation of the desorption energy method, additional TDS investigation of separation on time and temperature dependency, experimental results of desorption energy, theoretical selectivity calculation details, and computational error estimation (PDF)
