# Supplementary material for: Chemical Affinity of Ag-Exchanged Zeolites for Efficient Hydrogen Isotope Separation
Source: Inorg Chem. 2022 Jun 14;61(25):9413–20. doi: 10.1021/acs.inorgchem.2c00028 (PMC9241142; doi:10.1021/acs.inorgchem.2c00028)
Supplement: Supplementary file 1 — ic2c00028_si_001.pdf [file ic2c00028_si_001.pdf]

Supporting Information

Chemical Affinity of Ag Exchanged Zeolite for Efficient Hydrogen Isotope Separation

Linda Zhang[1*], Toshiki Wulf[2,3], Florian Baum[4], Wolfgang Schmidt[4], Thomas Heine[3,5], Michael Hirscher[1]

1 Max Planck Institute for Intelligent Systems, Heisenbergstr. 3, 70569 Stuttgart, Germany

2 Wilhelm-Ostwald-Institut für Physikalische und Theoretische Chemie, Linnéstraße 2, 04103 Leipzig, Germany

3 Helmholtz-Zentrum Dresden-Rossendorf, Forschungsstelle Leipzig, Permoserstraße 15, 04318 Leipzig, Germany

4 Department of Heterogeneous Catalysis, Max-Planck-Institut für Kohlenforschung, Kaiser-Wilhelm-Platz 1, D-45470 Mülheim an der Ruhr, Germany

5 TU Dresden, Fakultät für Chemie und Lebensmittelchemie, Bergstraße 66c, 01062 Dresden, Germany

Email: l.zhang@is.mpg.de

Thermal desorption spectroscopy (TDS)

TDS Procedure details for pure H_2 or D_2 desorption study. Firstly, the sample is activated in an ultrahigh vacuum (UHV) at 500 K, and then cool to RT. The sample is exposed to D_2 or H_2 atmosphere (10 mbar). Afterwards, the sample is rapidly cooled to the boiling temperature of the adsorbed gas, and the gas molecules that had not been adsorbed were pumped out. Finally, a linear heating ramp (0.1 $K \cdot s^{-1}$) is applied. The desorbing gas is continuously detected using a quadrupole mass spectrometer, recognizing a pressure increase in the sample chamber when gas desorbs. The area under the desorption peak is proportional to the desorbing amount of gas, which can be quantified after careful calibration (by $Pd_{95}Ce_5$ alloy) of the TDS apparatus.

TDS Procedure details for hydrogen isotope separation. The sample chamber is in an ultrahigh vacuum (UHV) at RT, and cool down to aimed temperature at 25 K, 40 K, 60 K, 77 K and 90 K, respectively. Then, the sample is exposed to a defined 1:1 D_2/H_2 mixture atmosphere (3, 10, 30 and 60 mbar) for 10 min. After 1:1 D_2/H_2 mixture loading at the given exposure temperature and pressure, the gas molecules that had not been adsorbed were pumped out. Afterwards, the sample is rapidly cooled to the boiling temperature of the adsorbed gas. Finally, a linear heating ramp (0.1 $K \cdot s^{-1}$) is applied in order to thermally activate desorption. The desorbing gas is continuously detected using a quadrupole mass spectrometer, recognizing a pressure increase in the sample chamber when gas desorbs. The area under the desorption peak is proportional to the desorbing amount of gas.

Calibration of the mass spectrometer signal. A solid piece of a diluted Pd alloy $Pd_{95}Ce_5$ (~0.5 g) was used for calibration. Before the calibration, the oxide layer of the alloy was removed by etching with aqua regia. Then the alloy was heated up to 600 K under high vacuum to remove any hydrogen that might be absorbed during the etching procedure. Afterwards, it was exposed to 40 mbar pure H_2 or pure D_2 for 1.5–2.5 h at 350 K after the mass had been collected. As H and D were bound preferentially to the Cerium atoms at low exposure pressures, the alloy could be

handled under ambient conditions for a short time. The alloy was weighed after being cooled down to room temperature. The mass difference between unloaded state and loaded state was equal to the mass uptake of hydrogen or deuterium, respectively. After weighing, the alloy was loaded in the chamber again, and then a 0.1 $K \cdot s^{-1}$ heating ramp (RT to 600 K) was applied for a subsequent desorption spectrum. The obtained mass of gas is directly corresponded to the area under the desorption peak.

Evaluation of the desorption energy

Desorption energy can be determined via *Kissinger* method[1] by employing desorption spectra recorded with different heating rates. After exposure to a 10mbar H_2 or D_2 pure gas at 60K the thermal desorption spectra have been recorded applying different heating rates of 0.01, 0.05, 0.1 and 0.2 $K \cdot s^{-1}$. The desorption maxima for both H_2 and D_2 show a clear shift to lower temperatures for slower heating rates indicating a thermally activated desorption process.

For an adsorbed gas molecule, the desorption process can be described by the Polanyi-Wigner equation for first order with the assumption that the pre-exponential factor and the desorption energy are independent of coverage:

$$-\frac{d\Theta}{dT} = \frac{\nu}{\beta} \cdot exp\left(-\frac{E_{des}}{RT}\right) \cdot \Theta \qquad \text{(Equation S1)}$$

where Θ is the referred to as the surface coverage, ν is the frequency factor, E_{des} is the activation energy for desorption, R is referred to the gas constant and T the measured temperature.

Considering the assumption that the surface coverage at the maximum temperature is independent of the heating rate, the temperature of the maximum desorption rate $T_{max} = T$ $(\max r_{des})$ is determined. The activation energy derivated from a plot $ln(T_{max}^2/\beta)$ vs. $1/T_{max}$, described as follows.

For $T = T_{max}$, the following condition must be fulfilled,

$$\left.\frac{dr_{des}}{dT}\right|_{T_{max}} = 0 \qquad \text{(Equation S2)}$$

Because of,

$$r_{des} = -\frac{d\Theta}{dT} = -\beta \cdot \frac{d\Theta}{dT} \qquad \text{(Equation S3)}$$

The equation 1 can be rewritten as

$$0 = \left.\frac{dr_{des}}{dT}\right|_{T_{max}} = n \cdot \Theta^{n-1} \cdot \frac{d\Theta}{dT} + \Theta^{n} \cdot \frac{E_{des}}{RT_{max}^2} \qquad \text{(Equation S4)}$$

For the first-order desorption process ($n = 1$), substituting $d\Theta/dT$ by equation 1, and solving for E_{des}/RT_{max}^2 obtains

$$\frac{E_{des}}{RT_{max}^2} = \frac{v_1}{\beta} \cdot exp\left(-\frac{E_{des}}{RT_{max}}\right) \qquad \text{(Equation S5)}$$

Desorption energy can be obtained from the slope by plotting ln $\left(\beta/T_{max}^2\right)$ vs. $1/T_{max}$ for a series of heating constant.

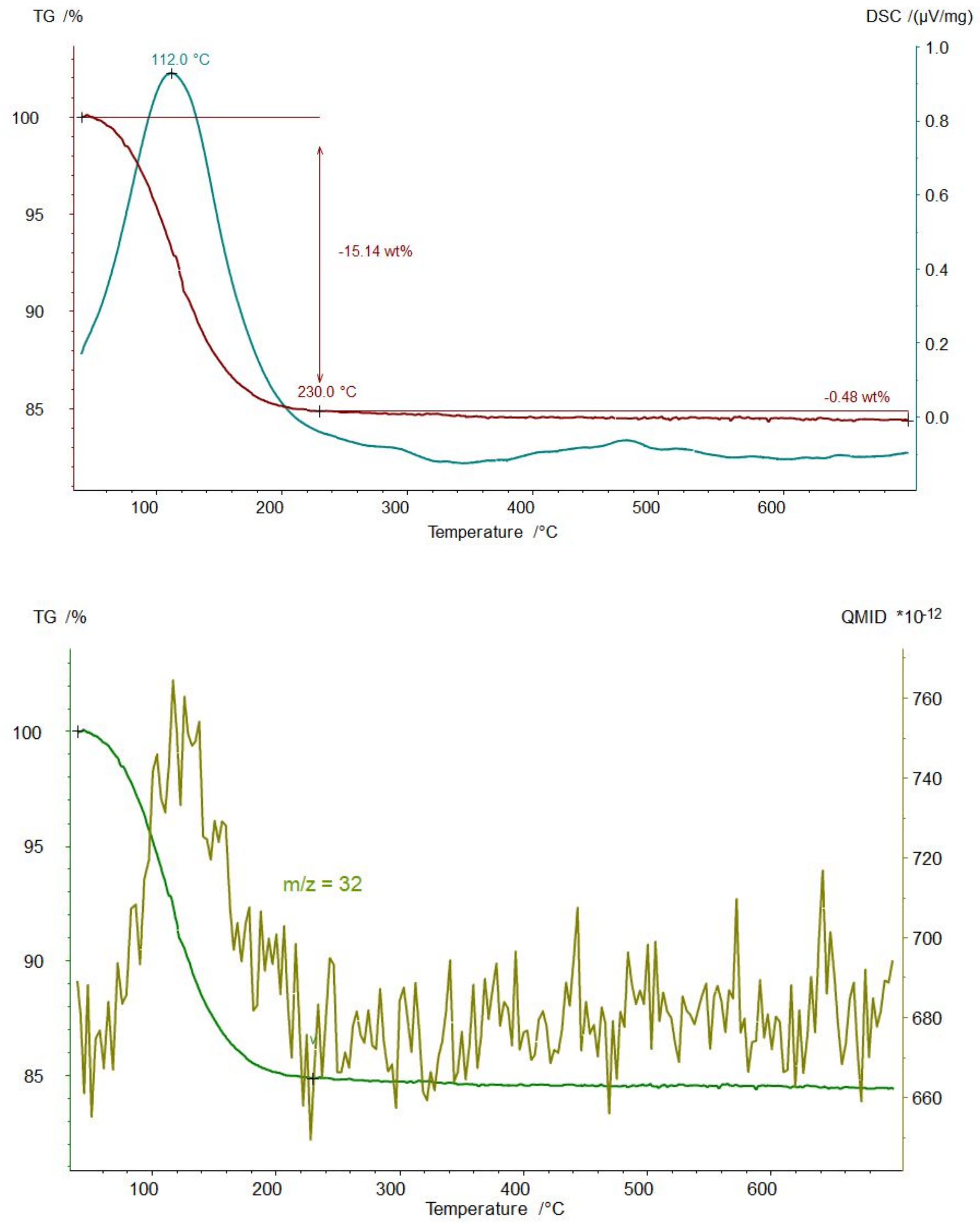

Figure S1. (Top) TG/DSC measurement of fully exchanged Ag-Y measured in argon flow. (Bottom) QMS signal of O_2 recorded simultaneously with the TG/DSC data.

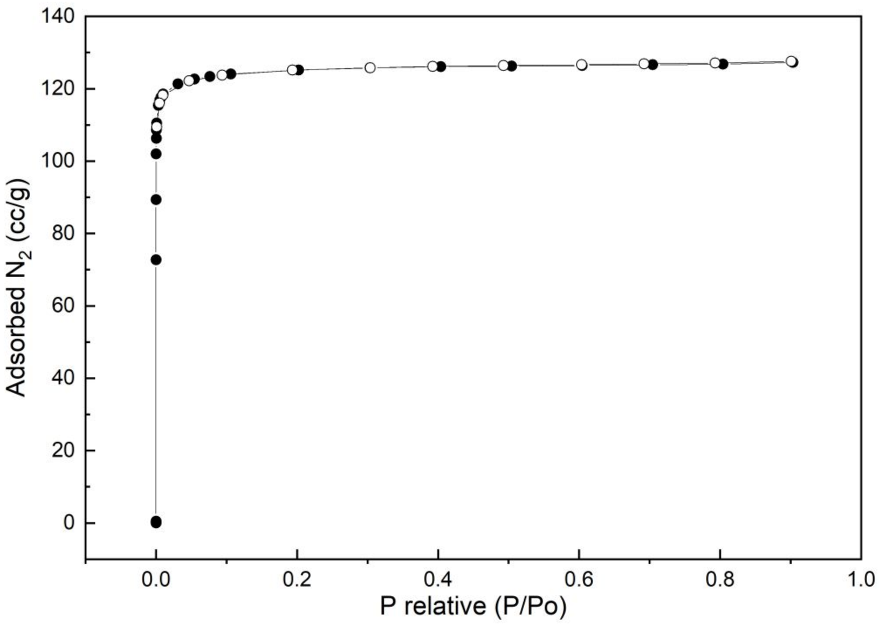

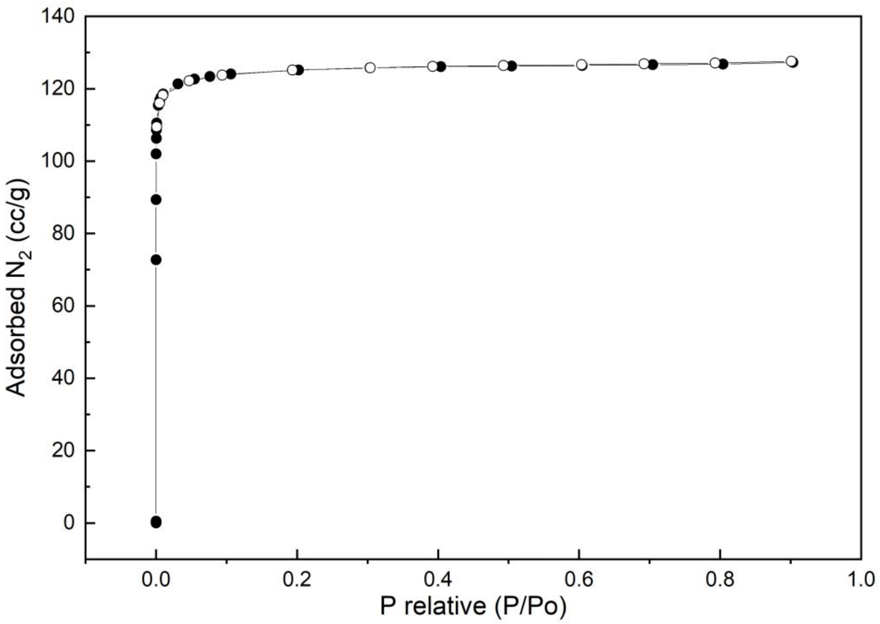

Figure S2. (Top) Nitrogen sorption isotherm of exchanged Ag-Y at 77K. (Bottom) Argon sorption isotherm of exchanged Ag-Y at 87K. Closed and open symbols represent the absorption and desorption of the sample.

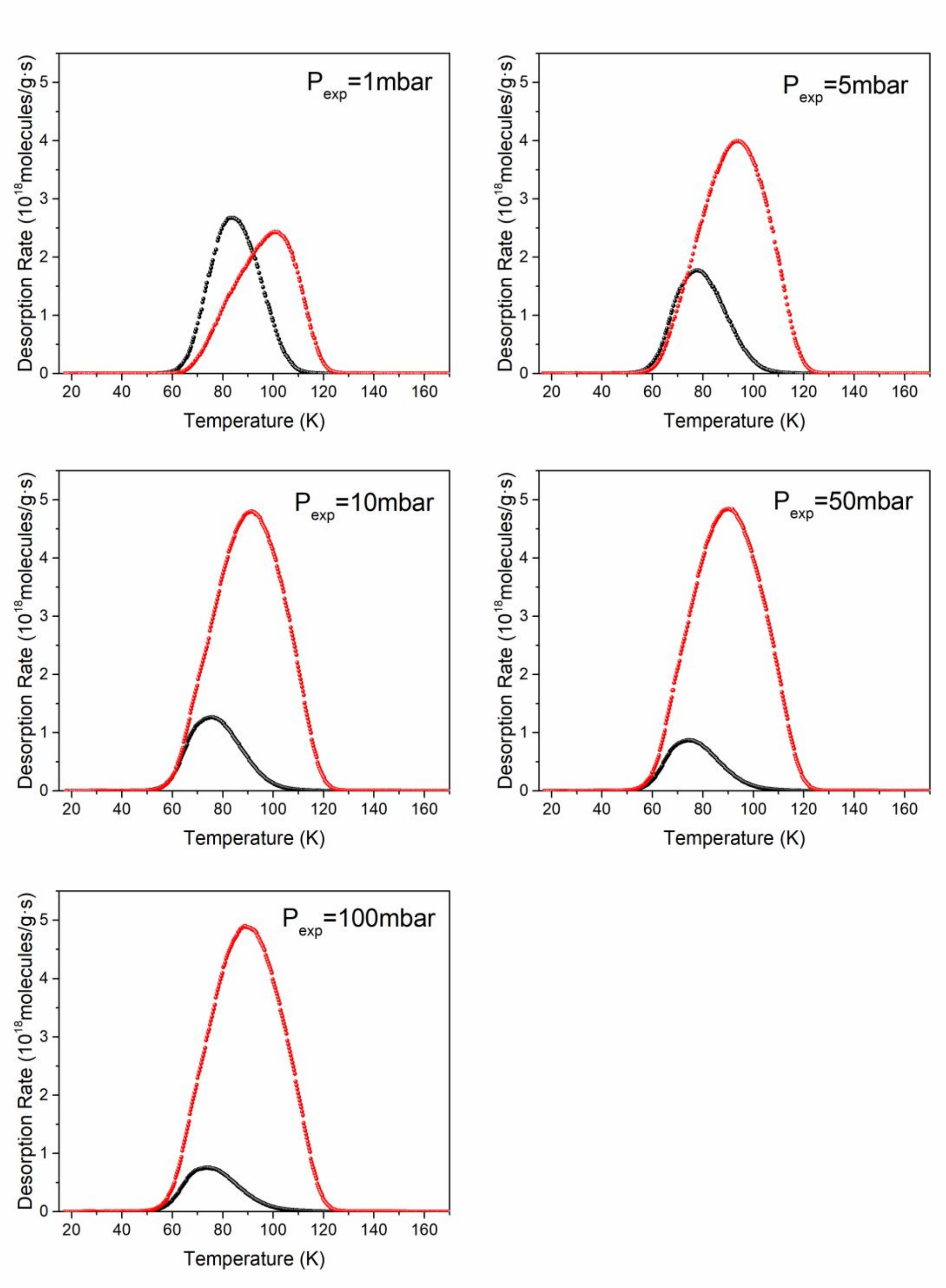

Figure S3. H_2 (black) and D_2 (red) desorption spectra of 1:1 D_2/H_2 mixture exposure on zeolite AgY at T_{exp} of 60 K for various exposure pressure (1, 10, and 100 mbar).

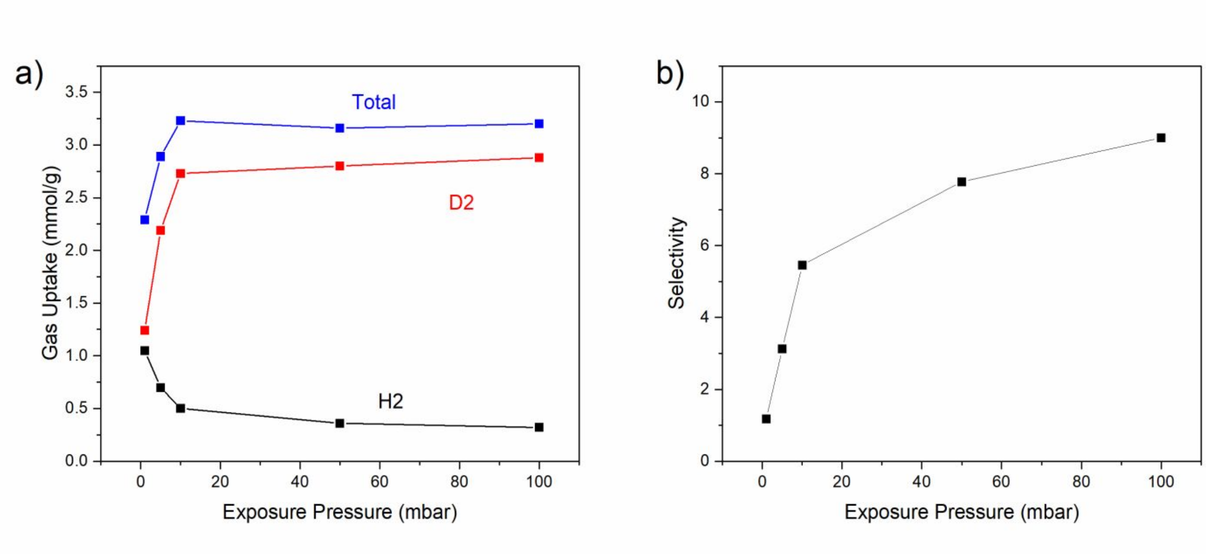

Figure S4. a) The adsorbed amount of isotope gases (H_2 black, D_2 red) and their total amount (blue) as function of exposure pressure obtained at T_{exp} of 60 K. b) The D_2/H_2 selectivity as function of exposure pressure.

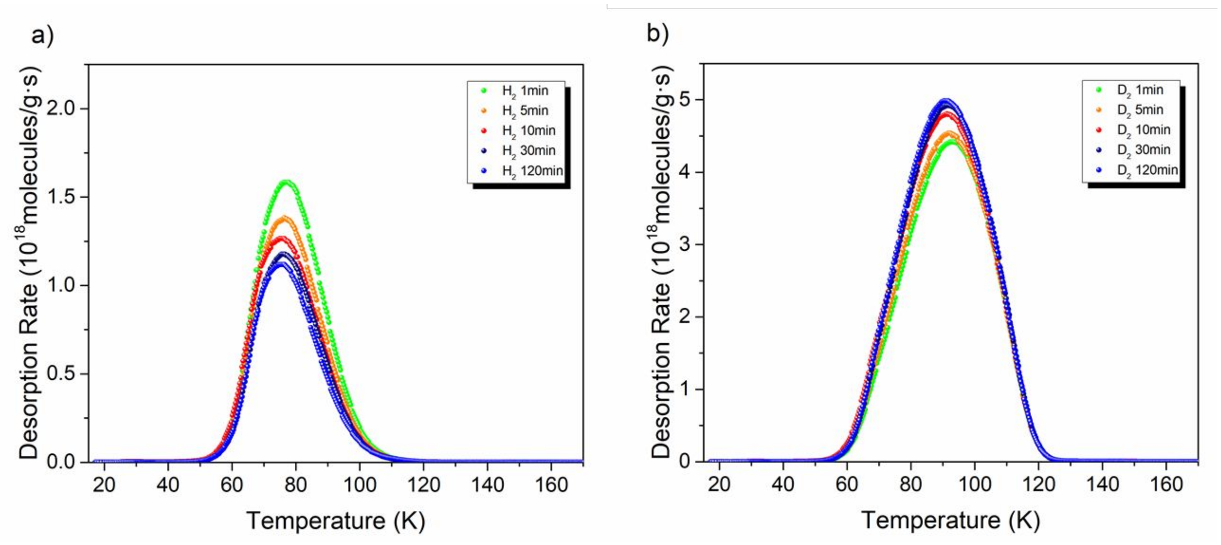

Figure S5. a) Hydrogen and b) deuterium desorption spectra of a 10 mbar 1:1 D_2/H_2 mixture exposure on zeolite AgY at exposure temperature of 60 K for various exposure time, 1 (green), 5 (orange), 10 (red), 30 (navy), and 120 (blue) min.

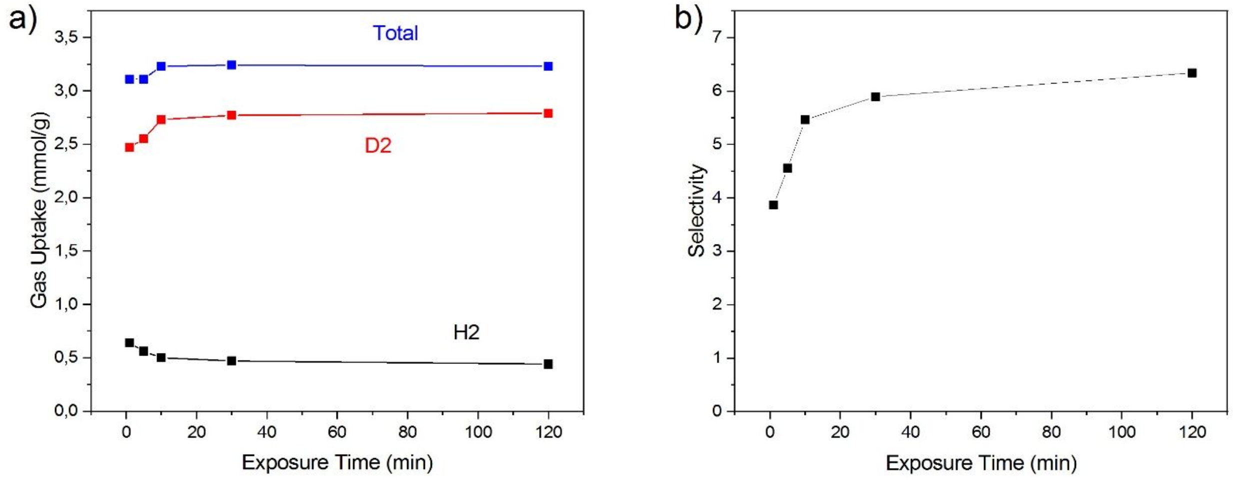

Figure S6. a) The adsorbed amount of isotope gases (H_2 black, D_2 red) and their total amount (blue) as function of exposure time obtained at T_{exp} of 60 K. b) The D_2/H_2 selectivity as function of exposure time.

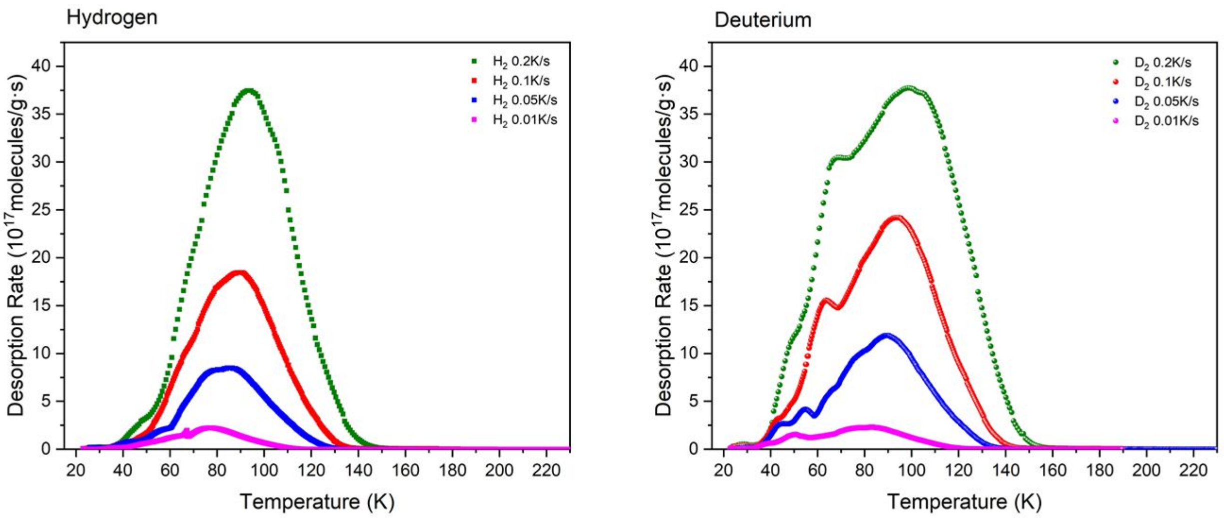

Figure S7. Thermal desorption curves for H_2 (left, square) and D_2 (right, circle) recorded at heating rates: 0.2 (olive), 0.1 (red), 0.05 (blue), 0.02 (green) K/s for zeolite AgY exposed to a 10 mbar gas atmosphere at 60 K. The minute shoulders/peaks on the low temperature side of the signals are likely caused by hydrogen/deuterium molecules adsorbed physically in cages of the zeolite Y framework at cryogenic temperature.

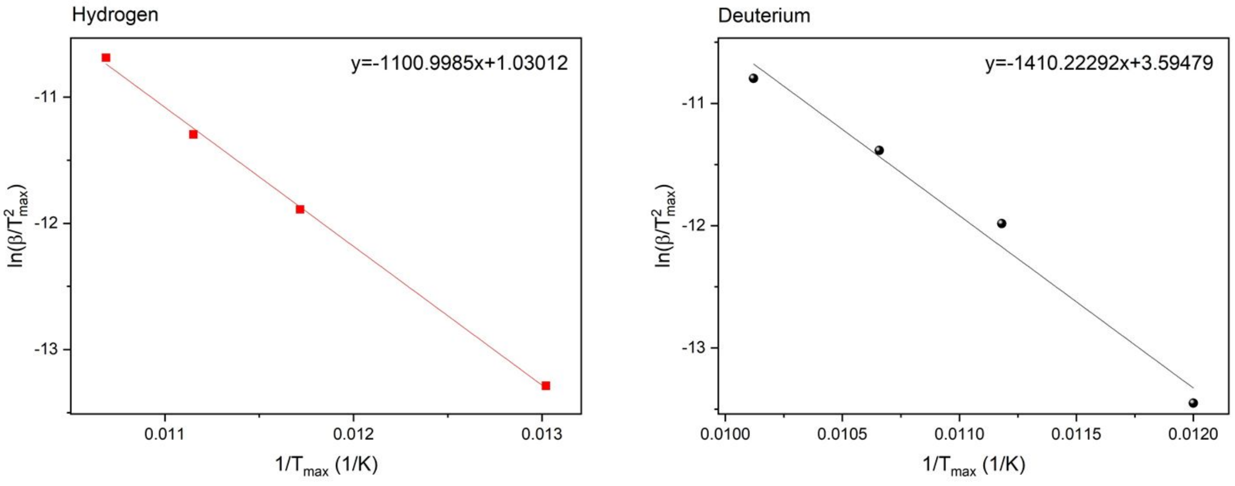

Figure S8. Kissinger plots for the desorption of H_2 (left, red) and D_2 (right, black) from AgY loaded at 60K.

Table S1. Summary of experimentally measured hydrogen isotope separation performance on various porous materials.

Compound	T_{exp}(K)	Selectivity (D_2/H_2) (1:1 Mixture)	Adosrbed D_2 amount (mmol/g)	Ref.
MFU-4 (Zn, Cl)	40	6.9	0.02	2
	50	5.8	0.40	
	60	7.5	1.24	
Py@COF-1	22	9.7	0.50	3
	30	7.9	0.60	
Cu(I)-MFU-4l	90	7.1	1.5	4
Fe-MOF-74	77	2.5	/	5
Co-MOF-74	77	3.2	/	
Ni-MOF-74	77	5	/	
ZIF-7	20	-	/	6
ZIF-8	20	11	/	
COF-1	20	7	/	
COF-102	20	1	/	
CPO-27-Co	60	11.8	2.7	7
IFP-1	30	2.0	9.30	8
IFP-3	30	2.8	2.49	
IFP-7	77	1.5	0.05	
IFP-4	77	2.1	0.01	
Zeolite 5A	30	2.7	4	9
CC3	30	1.7	3.67	10
	50	1.8	1.20	
6FT-RCC3	30	2.2	2.81	
	50	3.0	0.78	

**6ET-RCC3**	30	3.9	0.39	
	50	1.8	0.32	
**Cocryst1**	30	8.0	4.72	
**MIL-53(Al)**	40	10.5	2.3	[11]
**MOF-74**	77	19	3.5	[12]
**MOF-74-IM-10**	77	26	4	
**MOF-74-IM-38**	40	12.5	1	
**5A/GE2**	77	1.07	5.40	[13]
**CoFA**	25	16.6	5.5	[14]
**ZIF-67@NH_2–SiO_2**	77	1.52	/	[15]
**FMOFCu**	25	14	0.1	[16]
	77	4	0.05	
**Ag(I)-ZSM-5**	77	8.7	0.06	[17]
**Cu(I)-ZSM-5**	100	24.9	0.05	[18]
**SIFSIX-1-Cu**	20	7.1	0.1	[19]
**SIFSIX-3-Zn**	20	50	0.9	
**SIFSIX-3-Cu**	20	3.5	0.06	
**SIFSIX-3-Ni**	20	1.9	0.8	
**HKUST-1**	20	17	17.9	
**FCTF-1-400**	20	12.8	19.5	
**STAM-1**	20	9.9	2.8	
**Cu-PYC**	20	2.2	10.3	
**CPO-27(Co)**	20	3.0	13.8	
**KAUST-7**	20	9.8	0.37	
**FJI-Y11**	77	1.76	/	[20]
**Ni_2Cl_2BBTA**	77	4.5	1.7	[21]
**Ag(I)-Zeolite Y**	90	10	3	This work

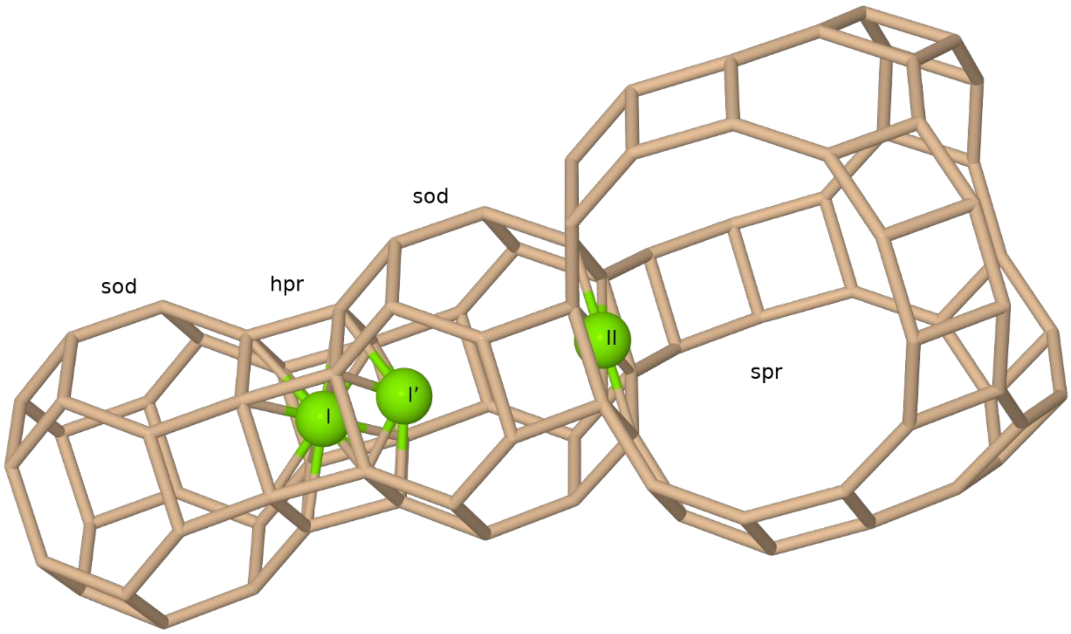

Figure S9. Cutout from zeolite Y framework showing main motifs including two sodalite cages (sod), a hexagonal prism (hpr) and a supercage (spr). Vertices signify T sites of the framework (Si or Al atoms); oxygen atoms omitted for clarity. Green spheres show extra framework cation sites I, I’ and II.

Computational errors

Treatment of vibrational modes. For the calculation of the thermodynamic properties, we use the harmonic approximation and only allow motion of the six degrees of freedom of the H_2 molecule. To validate this minimal approach, we compare it with a reference where only the terminal O–H groups are frozen in space and the rest of the cluster is allowed to vibrate. For these calculations, we use the generic models we have introduced in a previous publication[22] as well as versions of the generic model with one water molecule adsorbed at Ag^+.

A comparison of both approaches shows that the modes from the minimal approach have almost perfectly matching counterparts in the reference approach. The vibrational frequencies of corresponding modes agree within 20 cm^{-1} and the total error of the zero-point energy is less than 0.2 $kJ \cdot mol^{-1}$. The zero-point energy of the six modes of D_2 is underestimated, both in absolute terms and with respect to those of H_2, leading to a slight overestimation of the D_2/H_2 selectivity.

In the reference model, the remaining vibrational modes are almost identical irrespective of whether H_2 or D_2 is adsorbed at the Ag^+ site. Overall, these modes slightly favor the adsorption of D_2 over that of H_2, thereby cancelling out some of the above overestimation.

We conclude that the additional error incurred by the minimal vibrational treatment on the quantities relevant for the calculation of the selectivities, namely the *differences* between the adsorption energies of D_2 and H_2 should be very low and on the order of 0.1 kJ·mol^{-1}.

Computational level for single-point calculations. Previous experience[4, 23] shows that PBE0-D3(BJ) combined with typical triple- or quadruple-zeta basis sets is a robust method to estimate H_2 adsorption energies when the interaction is dominated by charge transfer effects. Ag-Y with an adsorption energy in the 30…40 kJ·mol^{-1} range would be considered to be such a system. However, the introduction of water leads to a considerable reduction of the H_2 adsorption energy for some structures and dispersion interactions make up a larger share of the total interaction energy. A correlated wavefunction approach, which natively includes dispersion interactions therefore becomes desirable and this is our rationale for resorting to the DLPNO-CCSD(T) method. While TightPNO cutoffs would be desirable for very weak interactions, these cutoffs are prohibitively resource-consuming for the systems at hand and the accuracy afforded by NormalPNO should be sufficient given the strength of the interaction.

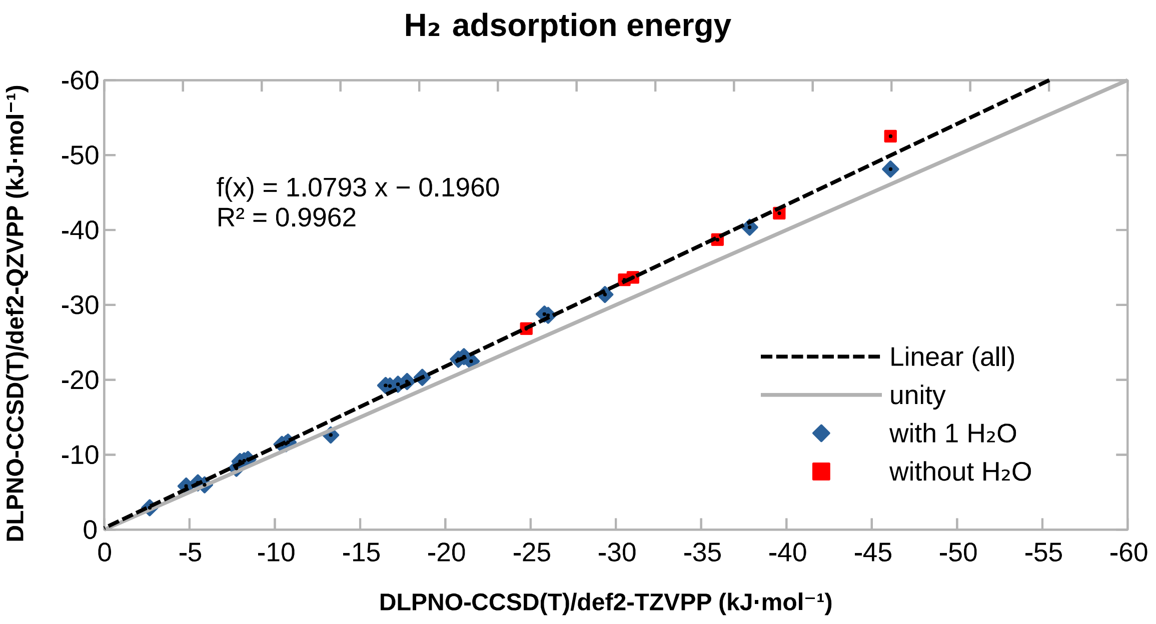

Figure S10. Comparison of DLPNO-CCSD(T) single-point energies for the adsorption of H_2 calculated with def2-TZVPP (x axis) and def2-QZVPP (y axis).

With NormalPNO cutoffs, the generous def2-QZVPP basis is still within reach for the systems with up to one molecule of water. Where possible, we compare this larger basis set with the more economical def2-TZVPP basis set. Like in prior studies we note that the differences between the reaction energies are small and def2-TZVPP predicts slightly weaker binding with a maximum deviation of +3.0 kJ·mol^{-1}, a minimum deviation of -0.6 kJ·mol^{-1} and average and root-mean-square deviations being -1.6 and 1.9 kJ·mol^{-1}, respectively (see Figure S10). We explain this effect with a comprehensive error cancellation between basis set superposition error (overestimating the

strength of the interaction) and basis set incompleteness error (underestimating the strength of the dispersion interaction), the latter apparently being slightly dominant. From a practical point of view, this justifies the use of def2-TZVPP.

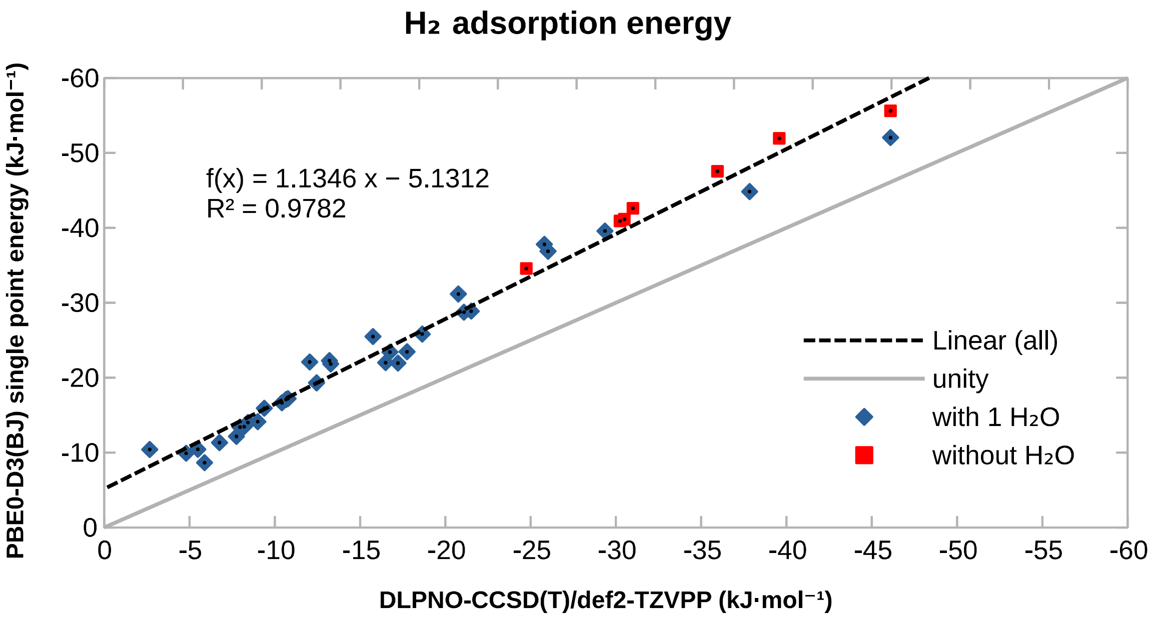

Figure S11. Comparison of single-point energies for the adsorption of H_2 calculated with DLPNO-CCSD(T)/def2-TZVPP (x axis) and PBE0-D3(BJ)/def2-QZVP (y axis).

Figure S11 shows the correlation between PBE0-D3(BJ)/def2-QZVP and DLPNO-CCSD(T)/def2-TZVPP single-point energies. It is apparent that the former overestimates the interaction by 5…10 kJ·mol^{-1} with respect to the latter. The error appears to be rather systematic,

so we expect this DFT method to be a robust predictor of trends for the system at hand where DLPNO-CCSD(T) becomes too expensive.

References

1. Kissinger, H. E., Variation of peak temperature with heating rate in differential thermal analysis. *Journal of research of the National Bureau of Standards* **1956,** *57* (4), 217-221.

2. Teufel, J.; Oh, H.; Hirscher, M.; Wahiduzzaman, M.; Zhechkov, L.; Kuc, A.; Heine, T.; Denysenko, D.; Volkmer, D., MFU-4–A Metal-Organic Framework for Highly Effective H2/D2 Separation. *Adv Mater* **2013,** *25* (4), 635-639.

3. Oh, H.; Kalidindi, S. B.; Um, Y.; Bureekaew, S.; Schmid, R.; Fischer, R. A.; Hirscher, M., A cryogenically flexible covalent organic framework for efficient hydrogen isotope separation by quantum sieving. *Angewandte Chemie International Edition* **2013,** *52* (50), 13219-13222.

4. Weinrauch, I.; Savchenko, I.; Denysenko, D.; Souliou, S.; Kim, H.; Le Tacon, M.; Daemen, L. L.; Cheng, Y.; Mavrandonakis, A.; Ramirez-Cuesta, A., Capture of heavy hydrogen isotopes in a metal-organic framework with active Cu (I) sites. *Nature communications* **2017,** *8* (1), 1-7.

5. FitzGerald, S. A.; Pierce, C. J.; Rowsell, J. L.; Bloch, E. D.; Mason, J. A., Highly selective quantum sieving of D2 from H2 by a metal–organic framework as determined by gas manometry and infrared spectroscopy. *Journal of the American Chemical Society* **2013,** *135* (25), 9458-9464.

6. Oh, H.; Park, K. S.; Kalidindi, S. B.; Fischer, R. A.; Hirscher, M., Quantum cryo-sieving for hydrogen isotope separation in microporous frameworks: an experimental study on the correlation between effective quantum sieving and pore size. *J Mater Chem A* **2013,** *1* (10), 3244-3248.

7. Oh, H.; Savchenko, I.; Mavrandonakis, A.; Heine, T.; Hirscher, M., Highly effective hydrogen isotope separation in nanoporous metal–organic frameworks with open metal sites: direct measurement and theoretical analysis. *Acs Nano* **2014,** *8* (1), 761-770.

8. Mondal, S. S.; Kreuzer, A.; Behrens, K.; Schütz, G.; Holdt, H. J.; Hirscher, M., Systematic Experimental Study on Quantum Sieving of Hydrogen Isotopes in Metal-Amide-Imidazolate Frameworks with narrow 1-D Channels. *Chemphyschem* **2019,** *20* (10), 1311.

9. Xiong, R.; Xicohtencatl, R. B.; Zhang, L.; Li, P.; Yao, Y.; Sang, G.; Chen, C.; Tang, T.; Luo, D.; Hirscher, M., Thermodynamics, kinetics and selectivity of H2 and D2 on zeolite 5A below 77K. *Micropor Mesopor Mat* **2018,** *264*, 22-27.

10. Liu, M.; Zhang, L.; Little, M. A.; Kapil, V.; Ceriotti, M.; Yang, S.; Ding, L.; Holden, D. L.; Balderas-Xicohténcatl, R.; He, D., Barely porous organic cages for hydrogen isotope separation. *Science* **2019,** *366* (6465), 613-620.

11. Kim, J. Y.; Zhang, L.; Balderas-Xicohténcatl, R.; Park, J.; Hirscher, M.; Moon, H. R.; Oh, H., Selective hydrogen isotope separation via breathing transition in MIL-53 (Al). *Journal of the American Chemical Society* **2017,** *139* (49), 17743-17746.

12. Kim, J. Y.; Balderas-Xicohténcatl, R.; Zhang, L.; Kang, S. G.; Hirscher, M.; Oh, H.; Moon, H. R., Exploiting diffusion barrier and chemical affinity of metal–organic frameworks for efficient hydrogen isotope separation. *Journal of the American Chemical Society* **2017,** *139* (42), 15135-15141.

13. Sun, N.; Li, P.-L.; Wen, M.; Song, J.-F.; Zhang, Z.; Yang, W.-B.; Zhou, Y.-L.; Luo, D.-L.; Zhang, Q.-P., Insights into heat management of hydrogen adsorption for improved hydrogen isotope separation of porous materials. *Journal of Materials Science & Technology* **2021,** *76*, 200-206.

14. Muhammad, R.; Jee, S.; Jung, M.; Park, J.; Kang, S. G.; Choi, K. M.; Oh, H., Exploiting the Specific Isotope-Selective Adsorption of Metal–Organic Framework for Hydrogen Isotope Separation. *Journal of the American Chemical Society* **2021,** *143* (22), 8232-8236.

15. Chen, X.; Liu, M.; Zhang, L.; Zhou, Y.; Ping, E.; Ding, C., New gas chromatographic packing ZIF-67@ NH2–SiO2 for separation of hydrogen isotope H2/D2. *Int J Hydrogen Energ* **2021,** *46* (24), 13029-13037.

16. Zhang, L.; Jee, S.; Park, J.; Jung, M.; Wallacher, D.; Franz, A.; Lee, W.; Yoon, M.; Choi, K.; Hirscher, M., Exploiting dynamic opening of apertures in a partially fluorinated MOF for enhancing H2 desorption temperature and isotope separation. *Journal of the American Chemical Society* **2019,** *141* (50), 19850-19858.

17. Xiong, R.; Chen, J.; Zhang, L.; Li, P.; Yan, X.; Song, Y.; Luo, W.; Tang, T.; Sang, G.; Hirscher, M., Hydrogen isotopes separation in Ag (I) exchanged ZSM-5 zeolite through strong chemical affinity quantum sieving. *Micropor Mesopor Mat* **2021,** *313*, 110820.

18. Xiong, R.; Zhang, L.; Li, P.; Luo, W.; Tang, T.; Ao, B.; Sang, G.; Chen, C.; Yan, X.; Chen, J., Highly effective hydrogen isotope separation through dihydrogen bond on Cu (I)-exchanged zeolites well above liquid nitrogen temperature. *Chem Eng J* **2020,** *391*, 123485.

19. Han, G.; Gong, Y.; Huang, H.; Cao, D.; Chen, X.; Liu, D.; Zhong, C., Screening of metal–organic frameworks for highly effective hydrogen isotope separation by quantum sieving. *Acs Appl Mater Inter* **2018,** *10* (38), 32128-32132.

20. Si, Y.; He, X.; Jiang, J.; Duan, Z.; Wang, W.; Yuan, D., Highly effective H 2/D 2 separation in a stable Cu-based metal-organic framework. *Nano Research* **2021,** *14* (2), 518-525.

21. Li, X.; Wang, X.; Li, M.; Luo, J.; An, Y.; Li, P.; Song, J.; Chen, C.; Feng, X.; Wang, S., Highly selective adsorption of D2 from hydrogen isotopes mixture in a robust metal bistriazolate framework with open metal sites. *Int J Hydrogen Energ* **2020,** *45* (41), 21547-21554.

22. Wulf, T.; Heine, T., Small crown-ether complexes as molecular models for dihydrogen adsorption in undercoordinated extraframework cations in zeolites. *The Journal of Physical Chemistry C* **2020,** *124* (17), 9409-9415.

23. Wulf, T.; Heine, T., Toward separation of hydrogen isotopologues by exploiting zero-point energy difference at strongly attractive adsorption site models. *International Journal of Quantum Chemistry* **2018,** *118* (9), e25545.
